# Exercise Intensity May Not Moderate the Acute Effects of Functional Circuit Training on Cognitive Function: A Randomized Crossover Trial

**DOI:** 10.3390/brainsci10100738

**Published:** 2020-10-14

**Authors:** Jan Wilke, Caroline Royé

**Affiliations:** Department of Sports Medicine, Goethe University Frankfurt, 60488 Frankfurt am Main, Germany; carolineroye@yahoo.de

**Keywords:** HIFT, cognition, neurocognition, effort, exertion

## Abstract

Functional circuit training (FCT) has been demonstrated to acutely enhance cognitive performance (CP). However, the moderators of this observation are unknown. This study aimed to elucidate the role of exercise intensity. According to an a priori sample size calculation, *n* = 24 healthy participants (26 ± 3 years, 13 females), in randomized order, performed a single 15-min bout of FCT with low (20–39% of the heart rate reserve/HRR), moderate (40–59% HRR) or high intensity (maximal effort). Immediately pre- and post-workout, CP was measured by use of the Digit Span test, Stroop test and Trail Making test. Non-parametric data analyses did not reveal significant differences between conditions (*p* > 0.05) although parameter-free 95% confidence intervals showed pre-post improvements in some outcomes at moderate and high intensity only. The effort level does not seem to be a major effect modifier regarding short-term increases in CP following HCT in young active adults.

## 1. Introduction

Many studies have established the beneficial effects of physical exercise on cognitive performance (CP, [1,2,3,4,5,6,7,8]). According to the available literature, chronic interventions (e.g., aerobic or resistance training), performed over weeks to months, enhance a variety of higher and lower order brain functions [5,6,7,8], which may be attributed to factors, such as enhanced cerebrovascular regulation, reduced systemic inflammation, improved insulin sensitivity or cortical neurogenesis [9,10,11]. Interestingly, also a single exercise bout can trigger improvements in specific domains, such as attention, working memory, inhibitory control and cognitive flexibility [1,2,3,4]. Acute increases in CP could be of interest in a variety of settings, including preparation for sporting activity or learning at schools and universities. However, the biological mechanisms and, even more, the optimal training parameters underpinning short-term gains in CP are obscure.

Aside from non-modifiable factors (e.g., age) which may also affect the effect magnitude [2,3,12], it has been proposed that exercise intensity represents a modifiable prime candidate driving immediate changes. Recent systematic reviews, however, differ in their conclusions. While some authors found light intensities most beneficial [2] others reported the largest effects at moderate [13], moderate to vigorous [4,5] exertion. Most studies examining the effects of exercise on CP focused on aerobic-type or resistance exercise. High-intensity functional training (HIFT) is a highly popular training method, which ranks among the top fitness trends worldwide [14] and aims to concurrently integrate cardiovascular and muscular efforts. Related workouts are based on the repeated execution of complex movement patterns (e.g., squats, lunges, push-ups) with minimal breaks in-between [15]. Data from intervention studies suggest that HIFT can acutely increase endurance capacity and muscle function [16], but beyond this, it also seems to enhance cognition. Following a 15-min bout of all-out circuit training, healthy individuals displayed improved short-term memory and inhibitory control [17]. This is of interest because the intensity spectrum for CP increases, so far, included light and moderate to vigorous but not maximal intensities [2,4,5,13]. The objective of the present trial, therefore, was to examine if changes in brain function following all-out HIFT, in fact, represent an outlier regarding previous knowledge or if functional circuit training performed at lower intensities would be comparably or even more effective.

## 2. Materials and Methods

### 2.1. Ethical Standards and Study Design

A three-armed, randomized, crossover trial, following the CONSORT (Consolidated Standards of Reporting Trials) guidelines [18] was performed in July 2020. It was prospectively registered at the German Register of Clinical Trials (DRKS00022285) and conducted in compliance with the Declaration of Helsinki. Ethics approval was granted by the local review board (2020-41, 14 July 2020, Ethics committee of the Faculty of Psychology and Sports Sciences, Goethe University, Frankfurt). Each participant signed informed consent. Enrolled individuals, in random order, completed three conditions: (1) functional circuit training at high-intensity (FCT-H), (2) functional circuit training at moderate intensity (FCT-M) or (3) functional circuit training at low intensity (FCT-L). Prior to and after the intervention, outcomes of CP were assessed. All participants visited the laboratory four times with seven-day intervals between the appointments. While the second to fourth visit included the actual experiments, the first was a familiarization session. Besides being introduced to the cognitive tests applied, participants received a demonstration of the functional circuit training workout.

### 2.2. Participants

Healthy adults (*n* = 24, 26 ± 4 years, 13 females) (Table 1) were recruited by means of personal addressing and poster advertising. All were physically active students engaging in 5 ± 2 sporting hours per week. The most performed types of exercise were fitness training in the gym and running. Exclusion criteria included (a) severe orthopaedic, cardiovascular, pulmonary, neurological, psychiatric or inflammatory rheumatic diseases, (b) pregnancy or nursing period, (c) analgesic intake during the trial or in the 48 h prior to study enrollment, (d) impairments in color vision, and (e) history of surgery or trauma in the lower extremity. Participants were asked to refrain from alcohol, caffeine, sugary drinks and strenuous physical activity during the 24 h preceding the three exercise sessions. To prevent influences of circadian rhythm, daytimes were kept constant within participants. Appointments were scheduled between 10 am and 2 pm (at least three hours after habitual wake-up time) as well as between 4 pm and 10 pm, as these intervals have been shown to be optimal for healthy individuals [19].

### 2.3. Intervention

The functional training intervention, performed at high intensity, has been shown to acutely enhance cognitive performance in a previous trial [17]. It consisted of 15 functional whole-body exercises performed in a circuit format with repeated 20s training bouts and 10s rest periods. At a total duration of 15 min, one workout thus had 30 exercise cycles. The selection of the exercises was based on two main goals: a) the concurrent activation of multiple major muscle groups to increase absolute oxygen consumption and b) the involvement of fundamental movement patterns mimicking activities of daily life (e.g., Squat, Lunge, Push-Up). Prior to the workout, a short general warm-up (rope skipping) was conducted.

The three exercise sessions differed with regard to exercise intensity. In FCT-H, the participants were encouraged to attain maximum workload (rather by increasing repetitions per bout than by increasing weights) while maintaining high movement quality. To facilitate the achievement of maximal workout intensity, music (140–160 beats per minute) was played [15].

In FCT-L and FCT-M, the participants performed an identical bout of functional circuit training but with light (20–39% of the heart rate reserve/HRR) and moderate (40–59% HRR) intensity, respectively [20]. To obtain HRR (HR_max_ − HR_rest_), we estimated HR_max_ by means of equation 208 (0.7 × age) [21] and measured HR_rest_ using electrical heart rate monitors (Beurer PM80, Beurer GmbH, Ulm, Germany) after being inactively seated for five minutes. Appropriate intensities were met by means of (a) reducing movement velocity and (b) offering modifications of the exercises (e.g., push-up on knees, use of lighter/heavier weights, such as medicine balls or rubber bands). Additionally, in these two conditions, music was played and constant feedback regarding exercise execution was provided to create an identical environment compared to the high-intensity workout. All interventions were supervised by a trained investigator with an academic degree in Sports Science. Session order was randomized by an investigator not involved in data collection using the software package “BiAS for Windows”, version 9.05 (Goethe-University Frankfurt, Frankfurt am Main, Germany).

### 2.4. Outcomes

Guided by the choice of tests in a previous study demonstrating CP improvements following FCT-H, we performed three assessments. The Stroop test measures aspects of attention and inhibitory control. In the word condition (S_w_), participants had to read black-inked words as fast as possible. In the color condition (S_c_), the same applied to naming colors. In the incongruent condition (S_cw_), words are presented in false colors (e.g., “green” written in red or “blue” written in yellow). Here, the participants had to name the color of the word while ignoring the letters. In all three parts, time until task completion was documented. The Stroop test has been demonstrated to display high reliability (Intraclass Correlation Coefficient/ICC: 0.82) and internal consistency (Cronbach’s alpha: 0.93 to 0.97) [22].

The Trail Making test (TMT) assesses attention, visual search and cognitive flexibility/working memory. In part A, disordered numbers have to be connected in ascending order using pen and paper (1 to 2 to 3 etc.). In part B, numbers and letters (e.g., 1 to a to 2 to b) were to be linked alternatingly. Similar to the Stroop test, time needed for completion was recorded. High reliability (ICC: 0.81 to 0.86) and construct validity of the TMT have been shown [23,24].

The Digit Span test has two conditions, which both measure short-term/working memory [25]. In the first, the participants need to memorize and repeat increasing amounts of numbers read to them. At the beginning, four numbers are to be recalled. In case of successful memorization, five numbers are named. For each step, two repetitions are performed, and one or zero points are awarded depending on recall success. The test ends if both trials are failed. The second condition is identical to the first, but numbers need to be repeated in reversed order (e.g., 2,4,7,9 becomes 9,7,4,2). The Digit Span test is reliable for repeated measurements (r = 0.73) [26].

Repeated assessments of cognitive function have been shown to be associated with practice effects [27]. We used two strategies to counteract this: 1) In the familiarization session, all individuals performed a series of tests until no further performance increments were noted; 2) different versions were used for each of the tests (two before and after each of the three sessions; six in total), which hence were never identical [27].

Besides cognitive performance, subjective arousal (Likert scale from "0—not activated" to "6—highly activated") and concentration (10 cm Visual Analogue Scale, 0—not concentrated at all to 10—highly concentrated) were assessed. After the interventions, the participants furthermore reported perceived exertion (6–20 RPE scale [28]) and exercise enjoyment (Likert scale from "0—not fun at all" to "6—most imaginable fun").

### 2.5. Data Processing and Statistics

The recruitment of the 24 participants was based on an a priori sample size calculation for a repeated measures ANOVA (F = 0.3, *p* = 0.05, power: 80%, drop-out 20%). Checks of sphericity (Mauchly’s test) and normal distribution (Kolmogorov–Smirnov test) revealed violations of the testing assumptions and hence, data were analyzed by means of non-parametric methods. We used the Friedman test to detect differences between conditions (FCT-L vs. FCT-M vs. FCT-H). For the detection of potential pre-post changes within the respective conditions (e.g., ∆ FCT-L baseline to FCT-L post), parameter-free 95% confidence intervals (CIs) were constructed. While classical CIs are based on the mean value, parameter-free CIs use the sample median and do not depend on data distribution. Their interpretation, however, is identical [29]. Calculations were made with “SPSS Statistics”, version 24 (IBM, SPSS Inc., Chicago, IL, USA) and “BiAS for Windows”, version 9.05 (Goethe-University Frankfurt, Germany).

## 3. Results

All individuals completed the study without the occurrence of dropouts. No baseline differences were found for arousal, concentration and cognitive baseline performance (*p* < 0.05; Table 2).

**Figure 1:**
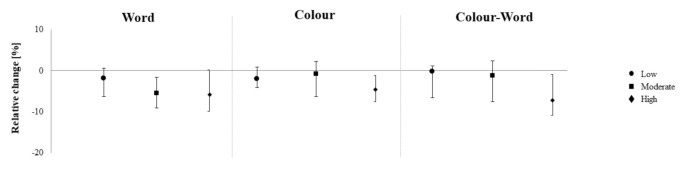
Pre-post differences in the Trail Making test as a function of exercise intensity. Figure shows medians and parameter-free 95% confidence intervals.

**Figure 2:**
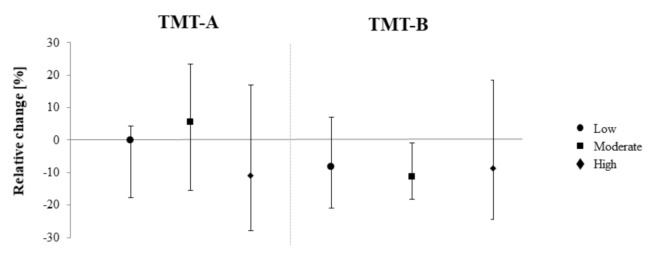
Pre-post differences in the Stroop test as a function of exercise intensity. Figure shows medians and parameter-free 95% confidence intervals.

Friedman tests did not reveal significant differences between the three exercise conditions (*p* < 0.05, Table 3). However, analysis of the 95% CIs suggested that both FCT-M (TMT-B: −11.59%, S_w_: −5.8%) and FCT-H (S_c_: −4.64%, S_cw_: −11.01%), contrarily to FCT-L, increased CP from pre to post in some outcomes (Figure 1 and Figure 2).

## 4. Discussion

During the last few decades, the role of exercise intensity has been a controversial topic in studies examining the acute cognitive effects of physical activity interventions [2,4]. While a large body of evidence is available for classical regimes, such as aerobic and resistance training, our trial is the first to address this issue in FCT. Contrary to our assumptions, no between-group differences were detected for the three tested effort levels. Therefore, we suggest that exercise intensity may be central for the achievement of motor function improvements [16], but does not seem to substantially drive CP changes.

If intensity would not represent a decisive effect modifier, the question arises which factors, in addition to the often-proposed quantitative variables (e.g., intensity, training duration) are intervening [30]. In a recent trial [31], we investigated the relevance of task complexity in resistance exercise. Interestingly, participants performing free-weight training, which requires higher levels of concentration and sensory motor coordination when compared to machine-based training, achieved larger improvements in executive function. Exercises performed in functional circuit training are very similar or sometimes, although performed with smaller or no weight, even identical to those in free weight training (e.g., squats, lunges, deadlifts [15]). We therefore suggest that the improvements following HIFT may be due to the complex nature of the exercises. Experiments using functional near-infrared spectroscopy and electroencephalography support this assumption. It has been demonstrated that complex motor activities lead to stronger cortical activations and larger oxygenation changes than simple tasks [32,33,34,35]. Against this background, future studies should test the hypothesis that the engagement in HIFT or free-weight exercise is linked to more complex cortical activation patterns when compared to simple or rather monotonous activities such as machine-based resistance training or aerobic exercise (e.g., cycling).

The present study has two major clinical implications. Firstly, as mentioned, exercise professionals should tie the selection FCT intensity to the goals of the intervention. If metabolic conditioning is targeted, the probable best solution is aiming to achieve high effort levels [16]. In contrast, if cognitive improvements are wanted, intensity may be sacrificed in favor of the introduction of complex exercises requiring high attentional demand and sensorimotor control. Nevertheless, secondly, if considering the acute CP effects of FCT in isolation, moderate or high intensity levels may still be preferable over light intensities. Although we failed to reveal differences between the three disposed conditions, analysis of the 95% confidence intervals suggested that only the two higher intensities induced CP improvements.

Our study has some limitations. The small-magnitude difference between effort levels could not be detected, possibly owing to the use of non-parametric data analyses exhibiting slightly lower power than parametric methods. Furthermore, we decided not to recruit a control group because the general effectiveness of FCT-H in increasing CP had already been demonstrated and our focus was to compare different exercise intensities. As a result, it cannot be ruled out that the observed pre-post changes were practice effects originating from repeated testing, although we made a strong effort to prevent them. Finally, another issue relates to the cognitive assessments itself. Our tests mainly captured lower-order executive functions, such as inhibitory control, cognitive flexibility or working memory. It can hence not be judged if different intensities in FCT would more strongly moderate changes in higher-order executive functions, such as problem solving or planning. Future studies may therefore consider expanding or modifying the choice of tests. Another call for further research relates to the target population. We examined young active individuals. Although exercise intensity does not seem to represent a major effect modifier for CP improvements following functional circuit training here, this may be different in elderly persons or sedentary participants.

## 5. Conclusions

In young and active adults, exercise intensity does not affect the magnitude of CP improvements following FCT to a major degree, although moderate and high exertions may be most beneficial. Additional research further delineating the dominant factors modifying CP—i.e., brain activation patterns—is warranted.

## Figures and Tables

**Table 1:** Sample data.

Parameter	Value
Weight (kg)	70 ± 11
Height (cm)	174 ± 10
BMI	23 ± 2
Resting heart rate (bpm)	67 ± 9
Maximal heart rate (bpm)	189 ± 3
Perceived exertion during exercise (RPE scale)	L: 9 ± 2, M: 12 ± 2, H: 16 ± 2

kg = kilogram, cm = centimeters, bpm = beats per minute, RPE = rate of perceived exertion (6: no exertion to 20: maximal exertion), L = light intensity, M = moderate intensity, H = high intensity.

**Table 2:** Pre-intervention values of cognitive performance prior to the three sessions.

	Light	Moderate	High	*p* Value
Stroop Word (t)	25.1 (21.1/31.5)	25.8 (19.3/30.8)	25.7 (19.5/33.5)	0.72
Stroop Color (t)	36.3 (25.5/46.1)	34.6 (24.5/44.3)	35.6 (26.2/52.3)	0.13
Stroop Interference (t)	55.1 (36.9/60.2)	52.0 (39.1/65.4)	54.5 (36.7/70.7)	0.25
Trail Making Test A (t)	22.1 (15.0/44.5)	21.6 (13.0/44.5)	22.9 (13.9/60.6)	0.10
Trail Making Test B (t)	24.5 (11.1/44.8)	21.8 (12.6/47.2)	21.5 (12.5/48.2)	0.42
Digit Span Score (pts)	11.5 (5/19)	11.5 (5/20)	11.0 (5/22)	0.95

Table shows medians and range (minimum/maximum). t = time in seconds, pts = points.

**Table 3:** Absolute pre-post differences in cognitive measures as a function of exercise.

	Light	Moderate	High
Stroop Word (t)	−0.51 (−1.57 to 0.18)	−1.22 (−2.66 to 0.05)	−1.35 (−3.9 to 4.39)
Stroop Color (t)	−1.17 (−2.87 to 0.16)	−0.28 (−2.0 to 0.78)	−1.65 (−2.81 to −0.22)
Stroop Interference (t)	−0.15 (−3.78 to 0.65)	−0.71 (−4.21 to 1.49)	−3.72 (−6.31 to −0.38)
Trail Making Test A (t)	0.11 (−4.05 to 6.09)	2.64 (−3.05 to 6.48)	−1.46 (−8.43 to 4.36)
Trail Making Test B (t)	−2.71 (−5.78 to 1.87)	−2.56 (−6.75 to −0.93)	−1.80 (−8.44 to 3.13)
Digit Span Score (pts)	0 (−2 to 1.75)	0 (−4 to 5)	−0.5 (−1 to 1.75)

Table shows medians and interquartile range. t = time in seconds, pts = points.

## Data Availability

Data will be made available on request.
